# TFEB Biology and Agonists at a Glance

**DOI:** 10.3390/cells10020333

**Published:** 2021-02-05

**Authors:** Mingyue Chen, Yashuang Dai, Siyu Liu, Yuxin Fan, Zongxian Ding, Dan Li

**Affiliations:** Collaborative Innovation Center of Yangtze River Delta Region Green Pharmaceuticals, Zhejiang University of Technology, Hangzhou 310000, China; mingyuechen0725@gmail.com (M.C.); daiyashuang09@gmail.com (Y.D.); liusiyu1102@gmail.com (S.L.); yuxinfandaisy@gmail.com (Y.F.); dingzongxian@gmail.com (Z.D.)

**Keywords:** TFEB agonists, autophagy, lysosome, rapamycin, resveratrol

## Abstract

Autophagy is a critical regulator of cellular survival, differentiation, development, and homeostasis, dysregulation of which is associated with diverse diseases including cancer and neurodegenerative diseases. Transcription factor EB (TFEB), a master transcriptional regulator of autophagy and lysosome, can enhance autophagic and lysosomal biogenesis and function. TFEB has attracted a lot of attention owing to its ability to induce the intracellular clearance of pathogenic factors in a variety of disease models, suggesting that novel therapeutic strategies could be based on the modulation of TFEB activity. Therefore, TFEB agonists are a promising strategy to ameliorate diseases implicated with autophagy dysfunction. Recently, several TFEB agonists have been identified and preclinical or clinical trials are applied. In this review, we present an overview of the latest research on TFEB biology and TFEB agonists.

## 1. Introduction

Autophagy occurs in all types of eukaryotic cells, which is a major cellular pathway for degradation of long-lived proteins and cytoplasmic organelles [1]. The degradation of proteins and organelles via autophagy plays an important role in maintaining cell homeostasis and responding to certain environmental stresses [2]. Many diseases, such as cancer and neurodegenerative diseases, are closely related to the failure of autophagy regulation mechanisms.

The transcriptional factor EB (TFEB) is a member of the microphthalmia-transcription factor (MiTF)/TFE family of leucine zipper transcription factors, a signal regulator to promote autophagy [3]. TFEB, generally isolated from the cytoplasm, will be translocated to the nucleus to coordinate the expression and regulation of lysosomes if activated. It is considered the main activator of autophagy-lysosomal gene expression [4]. Hence, TFEB agonists are emerging as novel therapeutic strategies to the treatment of diseases with autophagy-lysosomal dysfunction. The regulation mechanism of TFEB is a very complex process, and the research on TFEB agonists is increasing. So far, TFEB agonist, such as rapamycin, has been approved for the treatment of cancer [5]. Many TFEB agonists are still in preclinical trials. This review mainly elaborates on TFEB agonists and TFEB biology to contribute to the latest TFEB research trends.

## 2. Autophagy and the Lysosome

Autophagy is a lysosomal-dependent degradation pathway characterized by cytoplasmic vacuolization. Autophagy degrades the damaged structures in the cytoplasm to produce amino acids and free fatty acids for protein and energy synthesis, so that cells can adapt to different environments such as hypoxia and starvation [6]. In mammalian cells, autophagy is divided into macroautophagy, microautophagy, and chaperone-mediated autophagy [1]. Under physiological conditions, autophagy activity is required to maintain the homeostasis of the system. For example, autophagy related 9 (ATG 9), the sole transmembrane protein, mediates lipid scrambling and plays a crucial role in the lipid transport system that enables phagophore expansion [7,8]. Additionally, ATG 16L1 contributes to cell survival in the nutrient depletion state [9]. Under pathophysiological conditions, autophagy will be upregulated as a protection mechanism. For example, autophagy can be activated by serum starvation treatment in endothelial cells. During early starvation, autophagy could protect endothelial cell barrier from breakdown by inhibiting the reactive oxygen species (ROS) production [10].

The survival or death of cells is determined by the degree of autophagy. Physiological levels of autophagy, generally at a relatively low level, promote survival, whereas insufficient or excessive levels of autophagy promote death [11]. Hence, regulating the level of autophagy has been a therapeutic method for diseases related to autophagy. Autophagy can be activated as autophagosomes in HepG2 cells treated with 1.0mg/mL matrine. Matrine can inhibit the proliferation of HepG2 cells in a dose-and time-dependent manner [12]. Apoptosis and autophagy in T-cell acute lymphoblastic leukemia cells can be induced by resveratrol [13]. Puerarin can inhibit hyperglycemia-induced NLRP3 inflammasome activation in endothelial cells via regulating autophagy processes [14]. Apoptosis induced by cisplatin can be enhanced by insulin in human esophageal squamous cell carcinoma EC9706 cells, which is related to the inhibition of autophagy [15].

Autophagy is a defense mechanism of cells against adverse environment stressors, but the failure of regulation mechanisms is closely related to many diseases. Autophagy in cancer cells shows a dual effect, which simultaneously inhibits effect and promotes cancer development. For instance, an ATG 9A dependent autophagic pathway also plays an important role in Beclin 2 negatively regulating tumor development [16]. But starvation-induced autophagy can promote the invasion and migration of bladder cancer cells [17]. Autophagy also sustains pancreatic cancer growth and promotes the survival of dormant breast cancer cells [18,19]. Therefore, reagents that may induce autophagy are immediate areas of cancer treatment research.

Autophagy participates in the degradation of abnormal proteins to prevent the accumulation in neurons. Hence, the occurrence of some neurodegenerative diseases, characterized by the change of intracellular protein degradation system, is related to autophagy. For example, the accumulation of synuclein proteins in Parkinson’s disease is related to the decrease of autophagy activity. Autophagy plays an important role in nutritional metabolism, blocking the process will lead to a series of metabolic diseases. For example, autophagy is necessary to maintain the structure, number, and function of islet β cells and plays a protective role under stress conditions [20,21]. Autophagy dysfunction will lead to decreased islet function [22]. For lipid metabolism, autophagy disorders can lead to the accumulation of cellular lipids, resulting in obesity, dyslipidemia, fatty liver, and other diseases [23].

Lysosomes, the cell’s recycling center, are acidic compartments filled with more than 60 different types of hydrolases in mammalian cells. Lysosomes are mainly responsible for breaking down endocytosis and autophagy substrates, such as membranes, proteins, and lipids, into their basic components. The digested products are transported out of lysosomes via specific catabolic derivatives or vesicles [24]. Lysosome-mediated signal pathways and transcriptional procedures can sense the state of cell metabolism and control the switch between anabolism and catabolism by regulating lysosomal biogenesis and autophagy [25]. Lysosomes also play a role in cellular metabolism, immunity, regulation of hormone secretion, and so on. For example, melanin secretion of melanocytes, bone tissue reabsorption by osteoclasts, proteolytic enzyme secretion of natural killer cells, regulation of cell membrane repair by calcium ions, antigen presentation by macrophages, and B lymphocytes are all related to the activity of lysosomes [26,27].

Pieces of evidence show that lysosomal dysfunction is related to a variety of human diseases. Lysosomal storage disease (LSD) is a group of metabolic diseases caused by lysosomal hydrolase gene mutations, lysosomal membrane protein mutations, or non-lysosomal protein mutations. These mutations affect catabolism, catabolic output or membrane transport, and lysosome function indirectly, respectively. A prominent feature of LSD is the primary and secondary excessive accumulation of undigested lipids in lysosomes, leading to lysosomal dysfunction and cell death, ultimately resulting in pathological symptoms of various tissues and organs [28]. Fabry disease, an inherited lysosomal storage disorder, is caused by a mutation in the *GLA* gene, which results in a deficiency of the α-galactosidase that is a lysosomal acid hydrolase. This deficiency causes multi-organ pathology with high morbidity and a reduced life expectancy [29]. Neuronal ceroid lipofuscinoses, characterized by a heterogeneous origin of the storage material, are caused by mutations in the *CLN3* gene, which encodes a multi-pass lysosomal membrane protein involved in lysosome homeostasis [30]. Other studies have shown that lysosomes are also associated with diseases such as gout, Gaucher’s disease, Parkinson’s disease, and cancer [31,32,33].

## 3. TFEB as a Drug Target

### 3.1. The Structure of TFEB

TFEB was first cloned from a human B-cell cDNA due to its capacity of binding the adenoviral major late promoter [34]. TFEB is a protein composed of 476 amino acid residues, mainly including glutamine-rich, helix-loop-helix, leucine-zipper, and proline-rich domains. TFEB pertains to the MiTF/TFE family of basic helix-loop-helix-leucine zipper transcription factors [35].

### 3.2. TFEB Function

Lysosomes undergo nutrient-sensitive adaptive changes in function and biogenesis [36]. Nearly all receptors serving lysosome biogenesis are under the transcriptional control of TFEB, a master regulator of the lysosomal system. It is discovered that there is a consensus DNA sequence in the promoters of 96 lysosomal genes, which is termed the coordinated lysosomal expression and regulation motif. It has been proved that TFEB specifically targets the Coordinated Lysosomal Expression and Regulation motif to up-regulate genes for lysosomal biogenesis and function [37]. TFEB coordinates the expression of lysosomal hydrolases, lysosomal membrane proteins, and autophagy proteins in response to pathways sensing lysosomal stress and the nutritional conditions of the cell among other stimuli [38]. TEFB also plays an important role in regulating lysosomal exocytosis [39]. Lysosomal exocytosis is a process by which lysosomal membrane fuses with the plasma membrane and the content of the lysosome is excluded from the cell. And the exocytosis of conventional lysosomes is regulated by Ca^2+^. Mucolipin 1 (MCOLN1)/TRPML1 is the principal Ca^2+^ release channel on the lysosomal membrane [36]. TFEB activates the calcium channel protein MCOLN1, which promotes calcium influx and the fusion of lysosome and plasma membrane [40].

TFEB is of great importance in various cellular physiological processes. The role of TFEB in regulating autophagy is dependent on the cellular localization of TFEB, which is controlled by its phosphorylation status. In nutrient-rich conditions, TFEB is phosphorylated and retained in cytoplasm. Upon starvation or under conditions of lysosomal dysfunction, TFEB is dephosphorylated and translocated from cytoplasm to the nucleus, where it is active to regulate the expression of target genes for cargo recognition, autophagosome formation, vesicle fusion, and substrate degradation. For example, TFEB overexpression activates the biogenesis of autophagosomes [41]. Also, it has been discovered that post-modification of TFEB contributes to its transcriptional activity. TFEB transcription activity is enhanced with histone deacetylase inhibitor suberoylanilide hydroxamic acid, which results in the activation of lysosomal function in human cancer cells [42]. MiTF has been proved to be subject to small ubiquitin-like-modifier modification, as were the related family members TFEB. Sumoylation affects TFEB transcriptional activity in a manner dependent on the promoter elements present in the target genes [43].

A series of in vivo roles of TFEB has been identified. TFEB regulates lipid breakdown in the liver via peroxisome proliferator-activated receptor γ coactivator 1 α (PGC-1α) and peroxisome proliferator activated receptor α (PPAR-α). TFEB and proper lysosomal function are necessary to endoderm differentiation [44]. There is a RANKL-dependent signaling pathway taking place in differentiated osteoclasts. It culminates in the activation of TFEB to enhance lysosomal biogenesis which is necessary for proper bone resorption [45]. Endothelial TFEB promotes glucose metabolism via upregulation of Insulin Receptor Substrate 1 and Insulin Receptor Substrate 2 [46]. TFEB plays an important role in resisting intestinal epithelial cell injury in vivo, potentially mediated by APOA1 [47].

## 4. Mechanisms of TFEB Activation

### 4.1. Ca^2+^-Dependent Mechanism

**Figure 1:**
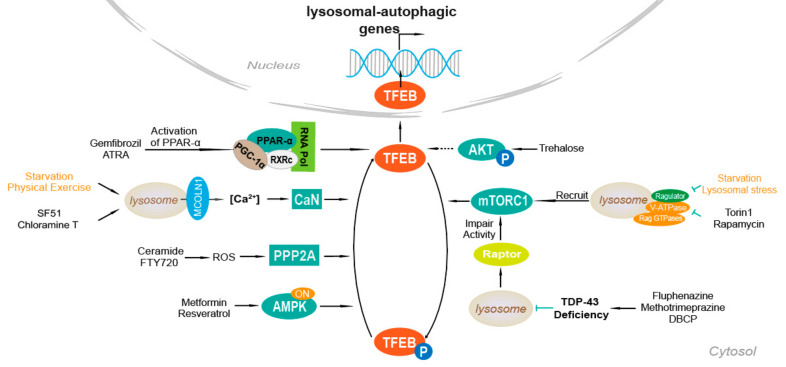
Schematic of mechanisms of TFEB agonists. The schematic shows the model of Ca^2+^ mediated regulation of TFEB, the model of lysosomal sensing and lysosome-to-nucleus signaling by TFEB and mTOR as well as the model of PP2A stimulating regulation of TFEB. AKT, loss of FLCN modulates the autophagy-lysosomal pathway via TFEB. PPAR-α-RXRα-PGC-1α complex and knocking down TDP-43 can promote the transcriptional activation of TFEB. Abbreviation: CaN, Calcineurin; AKT, protein kinase B.

There is a calcium signaling mechanism that begins with lysosomes and controls autophagy through calcineurin-mediated TFEB induction. Figure 1 shows a model that TFEB is regulated by mTOR- or calcineurin-related pathways under starvation or physical exercise. The lysosomal calcium channels on the lysosome membrane are involved in basic cellular processes. Recent discovery suggests that calcium microdomains probably locate on the surface of lysosomes. Calcium microdomains mediate local calcium signaling in several intracellular compartments, such as mitochondria [48].

The Ca^2+^ signaling mechanism of lysosome controls the activity of calcineurin phosphatase and TFEB. MCOLN1 is a direct transcriptional target of TFEB. There is a positive feedback loop in TFEB that promotes TFEB activity through Ca^2+^-mediated calcineurin activation by regulating MCOLN1 expression. After the release of lysosome Ca^2+^ through MCOLN1, the establishment of Ca^2+^ microdomains leads to a decrease in TFEB phosphorylation rate as mTORC1 is inhibited. TFEB dephosphorylation is induced by calcineurin, resulting in nuclear translocation of TFEB. Dephosphorylated TFEB is no longer able to bind 14-3-3 proteins and can freely move to the nucleus where it transcriptionally activates the lysosomal and autophagy pathway [49].

Furthermore, various oxidants including chloramine T, H_2_O_2_, and m-chlorophenylhydrazone can activate the MCOLN1/TRPML1, the major Ca^2+^ release channel on the lysosome membrane [36]. Additionally, the use of MCOLN1-specific agonist, SF51 can promote TFEB nuclear translocation in a calcineurin-dependent manner [49].

### 4.2. AKT

The serine and threonine kinase AKT, also known as protein kinase B integrates inputs from growth factors and metabolic effectors to control key multifunctional signaling hubs through direct phosphorylation of substrates that control cell growth, proliferation, and survival signal hub.

In the AKT-mediated autophagy-lysosomal pathway, AKT phosphorylates TFEB on S467, which is an evolutionarily conserved serine among vertebrate species and a close homolog of MiTF family. The TFEB phosphate mutant (TFEB S467A), in which the AKT phosphate receptor site is replaced by alanine to prevent AKT-mediated phosphorylation, increases nuclear localization and stability, and enhances the ability to activate TFEB downstream target genes. The use of pharmacological inhibitors or trehalose that inhibits AKT activity can promote nuclear translocation of TFEB and activation of the autophagy-lysosomal pathway, and enhance autophagy and lysosomal substrate clearance [50].

### 4.3. mTOR

The pathway regulating autophagy is mediated by calcium ion dependent phosphatase calcineurin. Figure 1 shows a model of TFEB regulated by mTOR or calcineurin-related pathways in the context of starvation and physical exercise. MTORC1 is a major kinase complex that plays an active role on the lysosome surface in mediating TFEB phosphorylation, positively regulating cell growth, and negatively regulating autophagy. MTOR mediated phosphorylation of TFEB-serine residues S142 and S211 promotes the interaction between TFEB and 14-3-3 protein, leading to cytoplasmic localization [51]. In contrast, conditions that lead to mTOR inhibition, such as starvation and lysosomal stress, promote TFEB nuclear translocation and transcriptional activation of lysosomal and autophagy genes.

The phosphorylation status of TFEB and its subcellular localization are entirely determined by the activation state of the Rag GTPases, which regulate mTORC1 activity downstream of amino acids. At any given time, some of TFEB rapidly and transiently binds to the surface of lysosomes, where phosphorylated and kept in the cytoplasm by mTORC1. When there are full nutrients without stress of lysosomal, the complex formed by V-ATPase, Regulator, and Rag GTPases is in the active state and recruits mTORC1 to the lysosomal surface, where mTORC1 is activated. At the lysosome, mTORC1 binds to and phosphorylates TFEB. TFEB then cycles between the cytoplasm and the surface of the lysosome. Under the phosphorylation conducted by mTORC1, TFEB remains in the cytoplasm and is prevented from being translocated to the nucleus. Starvation, v-ATPase inhibition, or lysosomal stress switches the Rags off, making mTORC1 depart from the lysosome and leading it to inactivation [52]. Hence, TFEB can be activated by Torin1, Rapamycin, and other inhibitors of mTOR.

### 4.4. PPAR-α Activation

Nuclear receptor peroxisome proliferator-activated receptor alpha (PPAR-α) is a PPAR subtype, a transcription factor that interacts with cis-acting DNA elements. PPAR-α agonists can induce the recruitment of the PPAR-α-RXRα-PGC-1α complex on the TFEB promoter and regulate the TFEB promoter region to enhance the transcriptional activation of TFEB, upregulating lysosomal biogenesis. After treating mouse primary astrocytes and neurons with gemfibrozil and ATRA in serum-free medium for 24 h, the overall level of TFEB is increased by 4 times, while the localization of TFEB in the nucleus is increased 5–6 times [53].

### 4.5. TDP-43 Loss of Function

TAR DNA-binding protein 43 (TDP-43) is considered a major component of the pathogenesis of ALS, FTLD, and other neurodegenerative diseases. TDP-43 regulates the localization of TFEB by targeting raptor, but not Rag GTPases. In TDP-43 knockdown cells, TFEB is translocated from the cytosol to the nucleus and formed lysosomal puncta in cytoplasm, whereas mTOR had a diffusively cytoplasmic distribution, suggesting involvement of mTORC1 in TDP-43-mediated TFEB nuclear translocation. Moreover, TDP-43-induced redistribution of TFEB and mTOR depends on raptor [54]. This regulation in turn enhances overall gene expression in the autophagy-lysosomal pathway (ALP) and increases autophagosome and lysosome biogenesis. However, the loss of TDP-43 also impaired the fusion of autophagosomes and lysosomes through the down-regulation of dynein 1, resulting in immature autophagic vesicle accumulation and overwhelming ALP function. Fluphenazine, methotrimeprazine, and 10-(4′-(N-diethylamino)butyl)-2-chlorophenoxazine (DBCP) have been proved to decrease TDP-43 protein level and activate autophagy in a live cell autophagic flux assay [55].

### 4.6. AMPK, FLCN, ERK

AMP-activated protein kinase (AMPK), a heterotrimeric enzyme, is an evolutionarily conserved energy sensor that functions to maintain energy homeostasis through coordinating effective metabolic responses to reduced energy availability [56,57]. AMPK not only elicits acute metabolic responses but also promotes metabolic reprogramming and adaptations through regulation of specific transcription factors and coactivators [56]. Folliculin (FLCN) is a binding partner negatively regulating AMPK. The interaction of FLCN with AMPK was regulated by two homologous FLCN-binding proteins FNIP1 and FNIP2. The ablation of FLCN expression or loss of the interaction of FLCN to AMPK causes constitutive AMPK activation, which is associated with metabolic transformation [56]. AMPK plays a pivotal role in regulating TFEB. Pharmacological activation of AMPK promotes TFEB localization, while phosphorylation status of mTOR shows no significant changes. The regulation of TFEB/TFE3 by FLCN is evolutionarily conserved and independent of mTOR pathway [57].

It has been shown that drugs including metformin and resveratrol activate AMPK indirectly by increasing cellular AMP and ADP through inhibiting mitochondrial ATP synthesis [58]. AMPK can also regulate extracellular signal-regulated kinase (ERK) levels, and cells with reduced phospho-ERK levels have elevated phospho-AMPK. Therefore, it can be concluded that inhibition of ERK can up-regulate TFEB [59].

### 4.7. PP2A Stimulation

Protein phosphatase 2A (PP2A) is a heterotrimeric enzyme with a scaffolding subunit “A”, regulatory subunit “B”, and catalytic subunit “C”. It is a major serine/threonine phosphatase that functions in regulation of many cellular processes including cell cycle, growth, metabolism, and apoptosis. Thus, PP2A is involved in many diseases including neurodegenerative disorders, cardiovascular pathologies, and cancer [60]. PP2A can dephosphorylate TFEB at several residues, including S109, S114, S122, and S211 in response to oxidative stress. This is a mTOR-independent pathway. Depletion of either PP2A catalytic subunits or the regulatory subunit, alone or in combination with the catalytic subunits, reduces TFEB-S211 dephosphorylation significantly. Ceramide or FTY720 can cause TFEB nuclear translocation through PP2A activation [61].

## 5. TFEB and Diseases

The current enzyme replacement therapy (ERT) for LSD has been proved successful in reversing cardiac abnormalities but has a limitation in skeletal muscle abnormalities [62]. Besides, the replacement enzymes are not easily accessible to target tissues. Compared to ERT, modulation of TFEB exploits lysosomal exocytosis to expel the storage material into the extracellular space, which solves the problem of enzyme delivery efficiency. TFEB is also proved to promote autophagosome-lysosomal fusion [40].

Pompe disease (PD) is a metabolic myopathy, characterized by the deficiency of acid alpha-glucosidase, which results in the excessive lysosomal glycogen storage. PD is also characterized by the secondary accumulation of autophagic debris. Overexpression of TFEB can stimulate fusion between lysosomes and autophagosomes, resulting in the formation of autolysosomes and increased exocytosis in muscle cells, in isolated live muscle fibers in PD mice [63,64].

Huntington’s disease (HD) is characterized by an expanded polyglutamine (PolyQ) chain in the HTT proteins, which is caused by the repeat expansion of CAG trinucleotide in the first exon of the *HTT* gene. PolyQ-expanded proteins misfold forming aggregates. Enhancing the autophagy-lysosomal pathway through TFEB overexpression can reduce HTT protein aggregation in cells. In a mouse model of HD, TFEB can improve neurological function when overexpressed [65,66].

Parkinson’s disease (PD) without fully defined pathogenesis has major pathological changes including the progressive death of nigral dopamine neurons and the accumulations of the pathogenic protein SNCA/α-synuclein [67]. The accumulation of SNCA invalidates the autophagy pathway, further leading to pathogenic protein aggregation [68]. In general, enhancing autophagy-mediated degradation of SNCA through TFEB regulation is a promising strategy for PD prevention and treatment.

Alzheimer’s disease (AD) is a neurodegenerative disease with main pathological features of β-amyloid peptides (Aβ) deposition in the brain and intracellular neurofibrillary tangle formation. In AD, the maturation of autophagosome-lysosome and retrograde transport are blocked, resulting in large swellings along dystrophic and degenerating neurites. The activated autophagy-lysosomal pathway is a key degradative pathway that can partially protect neurons from pathogenic protein aggregation [69].

## 6. Methods to Screen TFEB Agonists

There are plenty of methods to identify TFEB agonists. But there are no absolute criteria for determining autophagic status for each biological or experimental context. It is because some assays are inappropriate, problematic or may not work at all in particular cells, tissues, or organisms [70]. Table 1 shows the different methods to screen TFEB agonists. TFEB activation can be reflected by its subcellular localization. TFEB localization [71] can be directly revealed by experiments such as immunofluorescence. Real-time fluorescent quantitative PCR [72] shows that the expression of TFEB and TFEB downstream targets are up regulated after TFEB agonist treatment with high dynamic and accuracy. TFEB agonists can also be determined by the TFEB- promoter binding activity [53]. TFEB agonists are supposed to activate autophagy, and the change of autophagic flow can be detected by electron microscopy [73,74]. Therefore, the activity of TFEB agonists can be determined. When TFEB is activated, the protein expression of TFEB targets is upregulated, which can be assessed by Western Blot [75]. The electrophysiology technique [76] can measure the transmembrane current on phospholipid membranes to verify the effect of TFEB agonists as well. A nanotechnology-enabled high-throughput screen (HTS) [77] to identify small-molecule agonists of TFEB has been reported. It is an experimental method based on both cellular and molecular levels, so it has the characteristics of trace, rapid, sensitive, and accurate.

## 7. TFEB Agonists

TFEB agonists that have been discovered so far are shown in Table 2.

### 7.1. Direct TFEB Agonists

#### 7.1.1. Resveratrol

Resveratrol (3,4′,5-trihydroxystilbene, RSV) is a natural polyphenolic compound commonly extracted from grapes. In the study, RSV pretreatment in PA-treated human umbilical vein endothelial cells efficiently up-regulates LC3-II expression and down-regulates p62 expression, which indicates that RSV activates autophagy. It is proved that TFEB is partly a target of RSV. RSV promotes TFEB translocation into nucleus, evaluates TFEB and TFEB-downstream gene expression and thus promotes autophagy. It is clear that the expression of TFEB, lysosomal associated membrane protein 1 (LAMP1), and downregulating of p62 were significantly inhibited by siTFEB transfection in PA-treated human umbilical vein endothelial cells [78].

#### 7.1.2. Curcumin Analog C1

A synthesized curcumin derivative termed C1 has been proved as a new direct TFEB agonist. C1 specifically binds to TFEB and promotes the TFEB’s entry to the nucleus. Interestingly, TFEB promotes phosphorylation of RPS6KB1 and mTOR, which confirms that C1 activates TFEB without inhibiting mTOR pathway. At the same time, it is found that C1 has almost no effects on the activity of mTOR related kinases, which play fundamental roles in regulating autophagy. It has been proved that TFEB is especially required when C1 promotes autophagy through the experiments in which key autophagy genes *Atg5* (autophagy related 5), *Becn1* (beclin 1) and *TFEB* in N2a cells were knocked down. C1 is of structural stability and good blood-brain barrier permeability, so it is a potential drug for neurodegenerative diseases [80].

The effects of C1 on three AD animal models, which represent beta-amyloid precursor protein (APP) pathology (5xFAD mice), tauopathy (P301S mice) and the APP/Tau combined pathology (3xTg-AD mice), were investigated. And C1 efficiently promotes autophagy and lysosomal activity through TFEB activation. Thus APP, APP C-terminal fragments (CTF-β/α), β-amyloid peptides, and Tau aggregates are reduced [81]. The function of C1 in AD models indicates that TFEB agonists can be a new therapeutic target for such diseases.

#### 7.1.3. Progestin R5020

Prolonged treatment with progestin R5020 upregulates autophagy in MCF-7 human breast cancer cells via a novel interplay between progesterone receptor B and TFEB [82]. In addition, R5020 enhances the co-recruitment of progesterone receptor B and TFEB to mutually promote the verification of TFEB. Once in the nucleus, TFEB induces autophagy expression and lysosomal genes, which enhances autophagy. Since R5020-induced autophagy is independent of Akt-mTOR signaling, it is speculated that R5020 may affect TFEB activation, thereby enhancing autophagy in MCF-7 cells [82]. So, progestin R5020 is one of the TFEB agonists.

#### 7.1.4. Potential TFEB Agonists

Starvation or calorie restriction can activate TFEB. There are three novel compounds that promote autophagolysosomal activity, including the clinically approved drug, digoxin (DG); the marine-derived natural product, ikarugamycin (IKA); and the synthetic compound, alexidine dihydrochloride, which is known to act on a mitochondrial target. They participate in the TFEB activation mechanism through three different Ca^2+^ sources and Ca^2+^. And all three compounds promote autophagy flux and activate TFEB in a dose-dependent manner.

These Ca^2+^ sources selectively participate in the global Ca^2+^-sensing CaMKKβ-AMPK pathway or the local Ca^2+^-sensing MCOLN1-calcineurin pathway to release “brake” (inhibition of mTORC1 through AMPK) and/or promote TFEB activation “promoter” (activation of TFEB phosphatase). DG-induced lysosomal calcium released through MCOLN1 depends on the activity of an uncharacterized Ca^2+^-reactive TFEB phosphatase. Alexidine dihydrochloride-induced TFEB activation is realized by the direct perturbation of the mitochondrial protein PTPMT1 and the ROS-dependent MCOLN1-calcineurin pathway. Unlike DG and alexidine dihydrochloride, IKA triggers conventional ER-mediated Ca^2+^ release to activate the CaMKKβ-AMPK pathway [77].

In animals, TFEB plays a key role in promoting lipid metabolism during starvation, at least in part through global transcriptional activation of PGC-1*α* and PPAR-*α* [83]. Consistent with physiologically relevant TFEB activation, DG, alexidine dihydrochloride, and IKA significantly improves oleic acid-induced lipid accumulation in human hepatocytes. Newly discovered small-molecule TFEB agonists reduce metabolic syndrome and prolong lifespan of the body, addressing aging and age-related diseases.

### 7.2. Indirect TFEB Agonists

#### 7.2.1. Torin1

Previous studies have shown that TFEB is regulated by mTORC1 [90]. However, TFEB regulation by mTORC1 is complex and cell context-dependent [91]. Nutritional deprivation leads to the inhibition of mTORC1, the reduction of TFEB phosphorylation, and the promotion of TFEB nuclear translocation and lysosomal gene expression. It is reported that mTORC1 phosphorylates TFEB on S211 and on S142 [52]. The phosphorylation of S211 by mTORC1 creates a high affinity binding site for YWHA protein, which leads to the cytoplasmic retention of TFEB. When mTORC1 was inhibited, S211 is dephosphorylated and TFEB enters the nucleus.

Torin1 is an mTOR inhibitor [84]. When there are abundant nutrients and growth factors, TFEB is mainly cytoplasmic. However, after treatment with Torin1, TFEB was dephosphorylated and mainly localized in the nucleus. Hence, Torin1 treatment can transform TFEB into a rapidly migrating form of low phosphorylation, changing the distribution of TFEB from the whole cell diffusion mode to almost all nuclear cells. This phenomenon has been observed in many cell types [92]. TFEB regulation via Torin1 has been attributed to the change of S211 phosphorylation. The mutation from S211 to A increases the TFEB nuclear translocation [93]. Intra-articular injection of Torin 1 can reduce degeneration of articular cartilage in collagenase-induced osteoarthritis (OA), at least partially by autophagy activation, and it actives TFEB by inhibiting mTOR [85].

#### 7.2.2. Rapamycin

Rapamycin is an inhibitor that interferes with the inhibition of the mTOR signaling pathway, so it is a TFEB agonist [94]. In TNF/ zVAD-treated cells, rapamycin increases nuclear localization of TFEB and promotes autolysosome formation in a TFEB-dependent manner [88]. Rapamycin also suppresses TNF/zVAD-induced RIP1-S166 phosphorylation and increases phosphorylation of RIP1-S320, which is an inhibitory phosphorylation site [88]. Since TFEB is phosphorylated before activation, when the inhibit phosphorylation sites are suppressed, TFEB is more likely to be activated.

#### 7.2.3. 3,4-Dimethoxychalcone

Caloric restriction mimetics (CRMs) are natural or synthetic compounds that mimic the health-promoting and longevity-extending effects of caloric restriction. CRMs provoke the deacetylation of cellular proteins coupled to an increase in autophagic flux in the absence of toxicity [86]. 3,4-Dimethoxychalcone (3,4-DC), a member of CRM, is a TFEB agonist. When added to several different human cell lines, 3,4-DC induces the deacetylation of cytoplasmic proteins and stimulates autophagic flux [86]. Unlike other well-characterized CRMs, 3,4-DC, requires TFEB- and TFE3-dependent gene transcription and mRNA translation to trigger autophagy [86]. 3,4-DC stimulates the translocation of TFEB and TFE3 into nuclei both in vitro and in vivo, in hepatocytes and cardiomyocytes. GFP-TFEB transfers from cytoplasm to nucleus when treated with 3,4-DC, which can be observed by immunofluorescence or western blotting [86]. Tuberous sclerosis complex (*TSC2*) knockout, an operation that leads to structural activation of mTOR, inhibits 3,4-DC-induced TFEB translocation. *TSC2* gene knockout eliminates 3,4-DC induction of p62 and LC3-II protein levels. Moreover, like Torin1, 3,4-DC induces dephosphorylation of TFEB at S211, which is consistent with its ability to inhibit phosphorylation of another mTOR substrate, P70S6K. In vitro and in vivo research identifies 3,4-DC as a novel TFEB/TFE3 agonist through mTOR inhibition [95].

#### 7.2.4. Fisetin

Fisetin is an organic flavonoid found in a variety of fruits and vegetables, including strawberries, mangoes and cucumbers. Initially, it was identified in a screening of flavonoids. Fisetin activates autophagy, as well as TFEB and Nrf2 [87]. The activation of autophagy including TFEB is likely due to fisetin-mediated mammalian target of mTORC1 inhibition since the phosphorylation levels of p70S6K and 4E-BP1 are decreased in the presence of fisetin. Indeed, fisetin-induced phosphorylated tau degradation is attenuated by chemical inhibitors of the autophagy-lysosomal pathway. To examine whether TFEB is involved in fisetin-induced decrease of phosphorylated tau, cytosolic and nuclear fractions were prepared from cortical cells treated with vehicle only 5 or 10 μM of fisetin because upon activation TFEB enters nucleus [87]. Increases in the level of TFEB were observed in nuclear fractions of cells treated with 5 or 10 μM of fisetin compared to control cells treated with vehicle only. And mRNA levels of TFEB-downstream genes such as *ATG9B* and *LAMP1* were significantly increased in both cortical cells and primary neurons treated with fisetin.

## 8. Clinical Trials and Preclinical Trials of TFEB Agonists

The use of animal models, both lower organisms and mammals, has been very helpful to further elucidate TFEB function. In a mouse model of diet-induced fatty liver disease, TFEB agonists including digoxin, ikarugamycin, and alexidine dihydrochloride have been shown to improve lipid metabolism and overcome insulin resistance. These molecules represent clues to the development of treatment strategies for metabolic syndrome, aging and age-related diseases [77]. Among Akt modulators, MK2206, which is currently undergoing preclinical and phase I and clinical studies, is an effective oral inhibitor of Akt [89,96]. Intraperitoneal injection of MK2206 leads to inhibition of Akt activity and to TFEB nuclear translocation in a mouse brain, which in turn promotes the up-regulation of lysosomal and autophagic genes. It provides the evidence of enhanced pharmacological activation of TFEB for autophagy-lysosomal pathway in vitro and in vivo [97].

For the clinical trials of resveratrol, taking a dose of up to 5 g/day within a month is safe and well tolerated. However, dose-related mild to moderate side effects occur, coupled with resveratrol’s ability to alter the activity of drug metabolizing enzymes, which leads to the dose limitation used in future studies to <1.0 g/day. In human, oral resveratrol can be effectively absorbed. These effects should be further studied to help determine the optimal dose for the next phase of clinical trials for clinical efficacy evaluation [98]. A new research containing sixteen clinical trials shows that resveratrol supplementation significantly increases Glutathione Peroxidase serum levels. Hence, further large prospective clinical trials are needed to confirm the effect of resveratrol supplement on oxidative stress markers [79].

Curcumin analog C1 activates TFEB by directly binding to TFEB and promotes its entry into the nucleus, without affecting TFEB phosphorylation or inhibiting mTORC1 and MAPK1/ERK2 activity. Curcumin analog C1 works on the homozygous human P301S tau transgenic mice and homozygous 3xTg mice, and is still in the preclinical trials [81].

Rapamycin has already been an approved drug named “Sirolimus”, which is used to treat cancer [5]. Besides, progestin R5020, 3,4-Dimethoxychalcone, fisetin and Torin 1 are still in the process of preclinical trials [82,84,86,87].

## 9. Conclusions

The identification of TFEB as a global regulation of genes involved in the lysosomal–autophagic pathway, has provided new insights into the therapeutic role of TFEB. Owing to the broad number of diseases that potentially benefit from promoting lysosome and autophagy function, modulating the activity of TFEB represents an appealing therapeutic target. Genetic modification of TFEB has shown protection effects in several animal disease models. However, long-term effects of such treatments have not been evaluated. Studying the role and TFEB agonists in diseases will help provide a new perspective for the treatment and the development of new drugs. In this review, we summarize currently identified TFEB agonists, however, the effects of these agonists in preclinical or clinical trials still requires further investigations.

## Figures and Tables

**Table 1:** Methods to screen TFEB agonists.

Method	Function	Detection Indicator	Reference
Nuclear translocation	When TFEB is activated, it is displaced into the nucleus, so it is possible to determine whether TFEB is activated by observing the nucleus translocation.	DAPI, GFP, FITC, EB	Najibi, et al., 2016 [71]
Gene expression (qPCR)	Fluorescence chemicals were used to measure the total amount of products after each PCR cycle	CT value	Bao, et al., 2016 [72]
Promoter activation	The initiation of transcription is the key stage of gene expression, and promoter activation increases the level of gene expression.	Target protein	Ghosh, et al., 2015 [53]
Autophagy flux	Observing the accumulation of autophagosomes can reflect the induction of autophagosomes and the reduction of autophagosome consumption	mRFP-GFP-LC3, LC3-I, LC3-II	Castillo, et al., 2013 [73] Wang, et al., 2009 [74]
Protein expression (WB)	TFEB is activated and the amount of target protein produced increases.	Target protein	Dai, et al., 2020 [75]
Electrophysiology	Membrane potential changes as substances cross cell membranes, and TFEB changes are observed by recording the electrical activity of cells in the body.	Transmembrane current, Action potential	Kosacka, et al., 2013 [76]
A UPS-enabled high-throughput screen	Trace, fast, sensitive, and accurate screening the TFEB agonists.	PH, LAMP1	Wang, et al., 2017 [77]

**Table 2:** TFEB agonists.

TFEB Agonists	Structure	Mechanism	Clinical Trials and Preclinical Trials	Reference
Resveratrol (D)	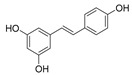	How RSV regulates autophagy has not been discovered.	Clinical trials	Zhou, et al., 2019 [78] Elnaz, et al., 2020 [79]
Curcumin analog C1 (D)	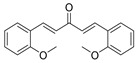	C1 binds specifically to TFEB, facilitating TFEB entry into the nucleus.	Preclinical trials	Song, et al., 2016 [80] Song, et al., 2020 [81]
Progestin R5020 (D)	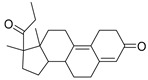	The interaction between PRB and TFEB increased autophagy in McF-7 breast cancer cells.	Preclinical trials	Tan, et al., 2019 [82]
Potential TFEB agonists (D)	A class of compounds related to Ca^2+^ that are structurally diverse	Ca^2+^ dependent mechanisms	Approved drug digoxin, ikarugamycin, alexidine dihydrochloride	Wang, et al., 2017 [77] Settembre, et al., 2013 [83]
Torin1 (In)	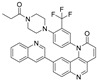	Inhibits mTOR	Preclinical trials	Thoreen, et al., 2009 [84] Cheng, et al., 2016 [85]
3,4-Dimet-hoxychalc-one (In)	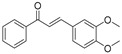	Inhibits mTOR	Preclinical trials	Chen, et al., 2019 [86]
Fisetin (In)	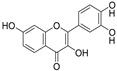	Inhibits mTOR and is related to ALP	Preclinical trials	Kim, et al., 2016 [87]
Rapamycin (In)	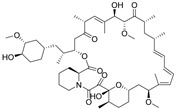	Inhibits mTOR	Approved drug: Sirolimus	Abe, et al., 2019 [88] Cui, et al., 2020 [89]

D = Direct TFEB agonists; In = Indirect TFEB agonists.

## Data Availability

Not applicable.
